# Visceral leishmaniasis on the Indian Subcontinent: Efficacy of fipronil-based cattle treatment in controlling sand fly populations is dependent on specific aspects of sand fly ecology

**DOI:** 10.1371/journal.pntd.0008011

**Published:** 2020-02-18

**Authors:** David M. Poché, Hsiao-Hsuan Wang, William E. Grant

**Affiliations:** 1 Genesis Laboratories, Inc., Wellington, Colorado, United States of America; 2 Ecological Systems Laboratory, Department of Wildlife and Fisheries Sciences, Texas A&M University, College Station, Texas, United States of America; Universidade do Estado do Rio de Janeiro, BRAZIL

## Abstract

**Background:**

Visceral leishmaniasis (VL) is a deadly disease transmitted by the sand fly *Phlebotomus argentipes* on the Indian subcontinent, with a promising means of vector control being orally treating cattle with fipronil-based drugs. While prior research investigating the dynamic relationship between timing of fipronil-based control schemes and the seasonality of sand flies provides insights into potential of treatment on a large scale, ecological uncertainties remain. We investigated how uncertainties associated with sand fly ecology might affect our ability to assess efficacy of fipronil-based control schemes. To do this, we used a previously-described, individual-based, stochastic sand fly model to quantify how uncertainties associated with 1) the percentage of female sand flies taking blood meals from cattle, and 2) the percentage of female sand flies ovipositing in organic matter containing feces from treated cattle might impact the efficacy of fipronil-based sand fly control schemes.

**Principal findings:**

Assuming no prior knowledge of sand fly blood meal and oviposition sites, the probabilities of achieving effective sand fly population reduction with treatments performed 3, 6 and 12 times per year were ≈5–22%, ≈27–36%, and ≈46–54%, respectively.

Assuming ≥50% of sand flies feed on cattle, probabilities of achieving efficacious control increased to ≈8–31%, ≈15–42%, and ≈52–65%. Assuming also that ≥50% of sand flies oviposit in cattle feces, the above probabilities increased further to ≈14–53%, ≈31–81%, and ≈89–97%.

**Conclusions:**

Our assessments of the efficacy of fipronil-based cattle treatments in controlling sand fly populations depend on our assumptions regarding key aspects of sand fly ecology. Assessments are most sensitive to assumptions concerning the percentage of sand flies ovipositing in feces of treated cattle, thus emphasizing the importance of identifying sand fly oviposition sites. Our results place the evaluation of fipronil-based cattle treatment within a broader ecological context, which could aid in the planning and execution of a largescale field trial.

## Author summary

Visceral leishmaniasis is a virulent disease transmitted to man by phlebotomine sand flies. Prior research has suggested the potential for fipronil-based drugs administered orally to cattle to successfully control adult sand flies feeding on cattle blood and larvae developing in cattle feces. However, field studies have yet to be conducted and uncertainties surrounding sand fly ecology may impact the success of treatment. We use an individual-based, stochastic model representing the sand fly life cycle, previously used to evaluate a number of potential fipronil-based sand fly control schemes, to investigate two of these uncertainties: 1) the percentage of sand flies feeding on cattle blood, 2) the percentage of sand flies laying eggs in cattle feces. Results indicate that changes in our assumptions regarding these two uncertainties have a significant impact on our estimates of the probable success of fipronil-based control schemes. Additionally, simulations suggest that the results are most sensitive to changes in the percentage of sand flies ovipositing in cattle feces. Hence, our results place the evaluation of fipronil-based cattle treatment within a broader ecological context and emphasize the need for novel approaches for determining the oviposition sites of sand flies.

## Introduction

Visceral leishmaniasis (VL) is a virulent vector-transmitted disease with an estimated 50,000 to 90,000 new human cases occurring worldwide each year, out of which only 25–45% are estimated to be reported [1, 2]. Over 60% of the reported human instances occur in poverty-stricken areas in India, Bangladesh, and Nepal [3]. On the Indian subcontinent, the known vector for *Leishmania donovani*, the causative agent of VL, is the sand fly species *Phlebotomus argentipes* [4, 5]. Although *L*. *donovani* is believed to be an anthroponotic pathogen with no known animal reservoirs [6], *P*. *argentipes* does feed on animal hosts and has been found to feed primarily on humans and bovines opportunistically [7–13].

Vector control in Bihar consists exclusively of indoor residual spraying (IRS), the practice of spraying the inner walls of homes and cattle sheds with insecticides. The impact of IRS on vector abundance has been highly inconclusive [12, 14–16], and its efficacy against sand flies is weakened by 1) the tendency of >95% villagers to sleep outdoors during the warmer months [17]; and 2) research indicating that a significant portion of *P*. *argentipes* may be arboreal and blood feed outdoors [11–13, 18–20]. Because the effectiveness of IRS is logically dependent on villagers and sand flies remaining indoors, alternative forms of sand fly control are needed to target outdoor vector populations. One such alternative may be the use of systemic insecticides (endectocides), which are used to control nematodes and arthropods affecting livestock [21]. One particularly promising compound is fipronil.

Fipronil is broad spectrum insecticide in the class phenyl pyrazole [22]. Fipronil acts by disrupting the central nervous system of insects by interfering with the passage of chloride ions through the GABA-regulated chloride channels (Rhone-Poulenc Ag Company 1996, now known as Bayer Crop Science). It has been shown to be highly effective as an oral endectocide against several disease vectors such as fleas [23–26], ticks [23], mosquitoes [27, 28], and *P*. *papatasi* [29, 30], *Phlebotomus mongolensis* [26] and *P*. *argentipes* [31, 32] sand flies. In terms of the efficacy of fipronil against sand flies, when administered to a host orally, it permeates in the blood and is excreted in the feces [33], allowing for control of both blood-feeding adult and fecal-feeding larval sand flies. A laboratory study in which fipronil-based grain baits were administered to rats (*Rattus rattus*, *Bandicotta bengelensis*) demonstrated that fipronil was more efficacious, faster acting, and had more prolonged efficacy against adult and larval *P*. *argentipes* relative to baits containing alternative insecticides (ivermectin, eprinomectin, diflubenzuron) [31]. Studies in which fipronil was administered orally to cattle (*Bos taurus*) under controlled conditions have demonstrated the potential for 100% control of *P*. *argentipes* adults and larvae for a minimum of 21 days after a single application [32]. Fipronil-based cattle-treatment may provide a promising new tool for sand fly control when performed at the village level. However, some important uncertainties in assessing efficacy under field conditions remain.

Poché et al. recently investigated the dynamic relationship between timing of fipronil-based sand fly control schemes and seasonality of the *P*. *argentipes* life cycle using an individual-based model parameterized to represent a village in Bihar, India [34]. These authors assumed that 1) *P*. *argentipes* females had a 50% probability of feeding on cattle blood [11], 2) gravid *P*. *argentipes* females had a 90% probability of ovipositing in organic matter containing feces from cattle [35], and 3) 100% of the village cattle would be treated. Considering cost-effectiveness and economic feasibility, the authors evaluated the performance of 20 simulated sand fly control schemes, in which the frequency and timings of treatments varied for each. The results suggested that applications performed at 2-month intervals, 3 times per year (March-July) and 6 times per year (January-November) were highly efficacious in reducing population peaks (≈90% and ≈95% reductions, respectively) as well as the cumulative number of sand fly days (≈83% and ≈97% reductions, respectively) occurring during peaks in VL incidence (April-August) and human exposure (June-August). Additionally, treatment conducted 12 times per year (monthly) led to eradication of the sand fly population within 2 years.

When administered orally, fipronil-based drugs remain active in cattle blood and are excreted in cattle feces, effectively targeting adult sand flies taking blood meals and larvae feeding on excreted feces. Thus, in addition to the control regime *per se* (timing of treatments and number of treatments) efficacy is dependent on the percentage of female sand flies 1) taking blood meals from treated cattle, and 2) ovipositing in organic matter containing feces of treated cattle. Yakob previously described a simulation model in which the success of systemic insecticide cattle treatment was dependent on two factors 1) the feeding behavior of the vector being targeted; and 2) the availability of alternative hosts [36]. Wang et al. also developed simulation models to assess the vector suppression treatments on cattle and found that host composition and population fluctuations influenced the outcome of treatments [37–40]. *P*. *argentipes* has been described as a “chance feeder”, feeding opportunistically on humans and cattle relative to host availability [10, 12]. The authors of Poché et al. hence indicated that the percentage of sand flies feeding on cattle implicitly represented the relative availability of cattle [34]. It seems logical that the encounter rate of sand flies with village cattle, relative to humans, might influence the blood feeding and oviposition tendencies of sand flies. Therefore, differences from village-to-village with regard to the density of livestock (host availability) and availability of cattle feces (oviposition sites) could significantly influence the percentage of sand flies feeding on cattle blood and ovipositing in cattle feces, and hence the efficacy of fipronil-based cattle treatment.

Recently, an application was submitted to the government of India for registration of a fipronil-based product to be orally administered to cattle for sand fly control [41]. If registration is awarded, an additional vector control technology will be available for use in villages in Bihar. Upon completion of the registration process, a large-scale field trial will aim to confirm the efficacy of fipronil-based application. In the meantime, ecological modelling serves as a means of providing a proof-of-concept by assessing efficacy of fipronil-based sand fly control in the face of current ecological uncertainties.

### Objectives

In this paper, we explore how uncertainties associated with sand fly ecology might affect our ability to assess efficacy of fipronil-based control schemes. By exploring these uncertainties, we aim to subsequently predict the outcome of vector control schemes implemented under a variety of ecological conditions representative of villages in Bihar. To do this, we relax model assumptions made by Poché et al. [34] and re-evaluate the three schemes they identified as most efficacious. More specifically, we use the model of Poché et al. [34] to quantify how uncertainties associated with 1) the percentage of female sand flies taking blood meals from cattle, and 2) the percentage of female sand flies ovipositing in organic matter containing feces from treated cattle might impact the efficacy of fipronil-based sand fly control schemes. This information could prove valuable for managers deciding whether to initiate treatment in candidate villages and at what frequency to do so.

## Materials and methods

### Overview of the simulation model

**Fig 1:**
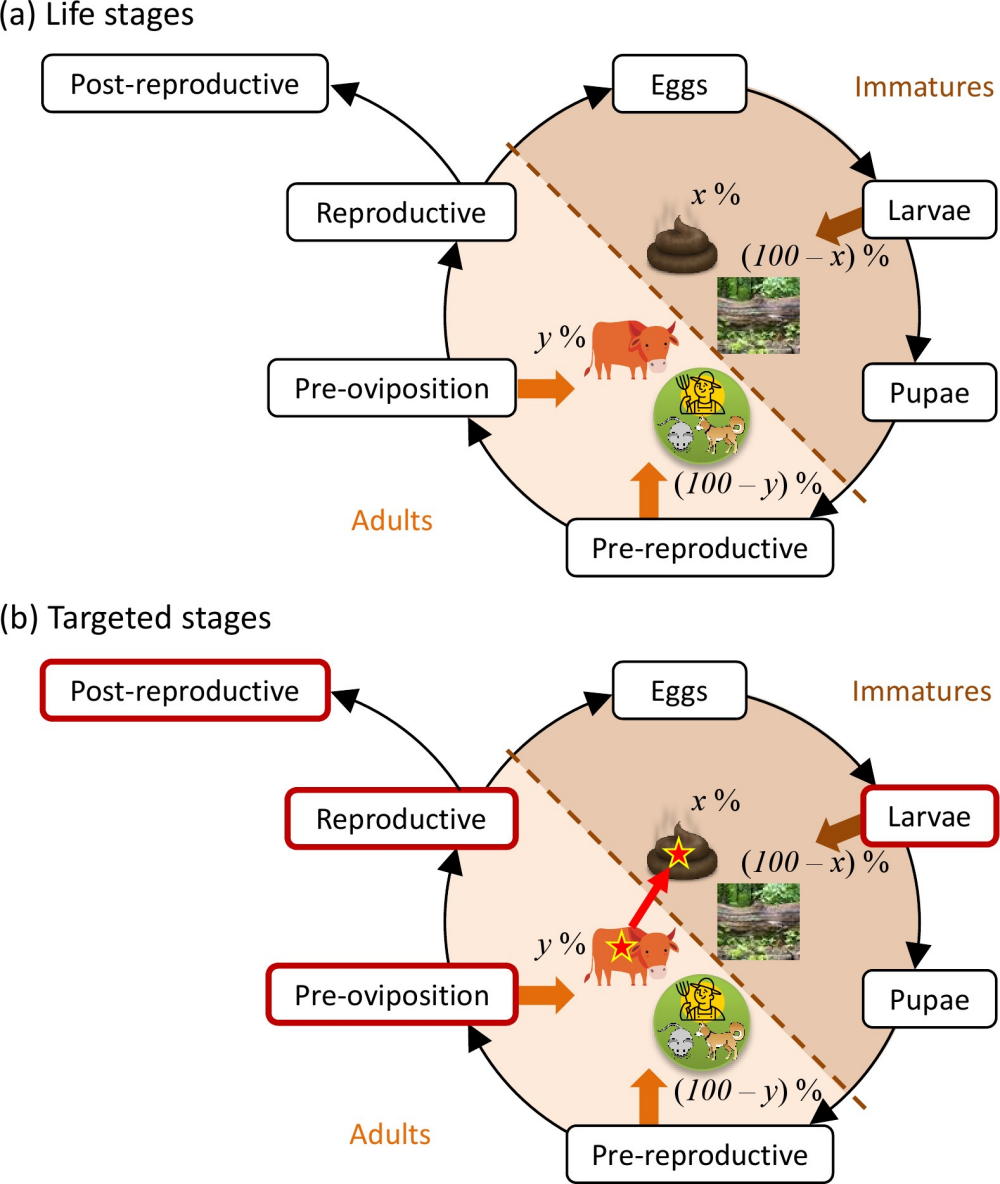
**Conceptual model representing (a) the sand fly life-cycle and (b) the life stages (outlined in red) targeted by fipronil-based control schemes.** Larval sand flies feed (brown arrows) on organic matter (brown shaded area), which may (*x*%) or may not ((100-*x*)%) contain cattle feces. Adult sand flies feed (orange arrows) on vertebrate hosts (orange shaded area), which may (*y*%) or may not ((100-*y*)%) include cattle. Stars represent the presence of fipronil, which is applied orally to cattle and subsequently passed (red arrow) in their feces.

The model, which is an adaptation of a model developed by Poché et al. [34] to evaluate vector control schemes in the Indian subcontinent, is individual-based and stochastic. The model simulates the effects of vector control schemes targeting cattle on the life cycle of sand flies in a village in Biha, India. The model represents the lifecycle of sand flies as they develop from eggs to larvae to pupae to pre-reproductive adults to pre-oviposition adults to reproductive adults to post-reproductive adults (Fig 1). Rates of development, natural mortality, and reproduction depend on the environmental temperatures to which the sand flies are exposed. Eggs, larvae, and pupae are exposed to temperatures of the organic matter in which they develop, whereas adults are exposed to ambient temperatures. Natural mortality of larvae also depends on the density of larvae in the organic matter in which they are feeding. Effects of vector control via fipronil-based drugs orally administered to cattle are represented by increasing mortality rates of (1) adult flies that obtain a blood meal from fipronil-treated cattle, and (2) larvae feeding in organic matter containing feces from fipronil-treated cattle. Efficacies of fipronil in cattle blood and in feces from treated cattle both decline exponentially after application of the drug [34]. Simulations are run for one year using a daily time step. Detailed model equations representing the development, reproduction, natural mortality, and fipronil-induced mortality of sandflies, as well as the data analyses involved in model parameterization, are available in Poché et al. [34].

### Experimental design for simulations

We evaluated uncertainty associated with assessing the efficacy of fipronil-based cattle treatment schemes involving different frequencies of application in view of the uncertainty associated with (1) the percentage of **a**dult sand flies **f**eeding on **c**attle (AFC) and (2) the percentage of reproducing **f**emales **o**vipositing in organic matter containing **f**eces from treated cattle (FOF). We focused on 3 specific schemes evaluated by Poché et al. [34]: (1) applications 3 times per year at 2-month intervals initiated in March, (2) applications 6 times per year at 2-month intervals initiated in January, and (3) applications 12 times per year at monthly intervals. The first 2 of these schemes were identified as the most efficacious, considering economic and logistical constraints. The third scheme represents an ideal management scenario. In all of their simulations, Poché et al. [34] assumed that all of the village cattle (100%) were treated, that 50% of the adult sand flies fed on cattle, and that 90% of the reproducing females oviposited in organic matter containing feces from treated cattle. We simulated several versions of each of the 3 schemes, varying the percentage of cattle treated (either 100% or 66.1%), as well as both AFC and FOF (from 0% to 100% in increments of 10%) (See the Appendix for verification that our current model produces the same results as the model of Poché et al. [34] under the conditions that they simulated.) Simulations assuming 100% of the cattle were treated allowed us to focus on the effects of uncertainties associated parameters representing sand fly ecology. Simulations assuming 66.1% of the cattle were treated allowed us to place our results within the context of a more realistic field situation [42].

For each variant of each scheme, we ran 10 replicate stochastic (Monte Carlo) simulations. During each simulation, the system was allowed to establish a dynamic equilibrium, without treatment, for 2 years, then treatment was applied annually for 3 consecutive years, during which time the abundance of adult sand flies was monitored. We assessed efficacy of each variant of each scheme during the third year of treatment based on (1) reduction of the **m**aximum daily number of **a**dult **s**and flies occurring April to August (MAS), (2) reduction of the cumulative number of adult **s**and fly **d**ays occurring **A**pril to **A**ugust (SDAA), and (3) reduction of the cumulative number of adult **s**and fly **d**ays occurring **J**une to **A**ugust (SDJA).

We summarized simulation results in the form of heat maps representing MAS, SDAA, and SDJA in which AFC was represented along the x-axis and FOF was represented along the y-axis. We superimposed isolines on the heat map surfaces that represented all combinations of AFC and FOF that produced selected combinations of MAS, SDAA, and SDJA. We also calculated percentages of heat map surface areas above and below these isolines, which represented probabilities of values higher or lower, respectively, than those represented by the points along the isolines. We constructed most heat maps under the assumption of a complete lack of prior knowledge regarding both AFC and FOF. That is, scales of both the x-axis and the y-axis ranged from 0% to 100%. However, in some cases, for purposes of illustration, we assumed prior knowledge of one or both percentages and restricted the scale of one or both of the axes accordingly.

### Application of results to other studies

We applied results of our simulations to Stauch et al. [43], WHO [44], Fitzpatrick et al. [45], and Sevá et al. [46] to suggest how explicit assessment of uncertainties associated with sand fly ecology might provide additional value to assessments of vector control needs. Each of these studies provided data which can be used to estimate the percent reduction in vector populations constituting efficacious control. Stauch et al. estimated that reducing sand fly populations by 67–72% would subsequently eliminate VL from a human population [43]. The WHO has proposed a global response to the 80% of the global population estimated to be at risk of vector-born disease [44]. Fitzpatrick et al. divided vector control technology into two broad categories and considered a “high-efficacy” technology to be one which reduced a vector population by 70–90% [45]. da Paixão Sevá et al. [46] simulated the impact of phlebotomine sand fly control during a cutaneous leishmaniasis outbreak in Madrid, Spain when the vector population was reduced by 75% [46]. Therefore, we used these values (67–72%, 80%, 70–90%, 75%) as a benchmark for efficacious vector population reduction during the current simulations. We calculated uncertainties that might be associated with these estimates of efficacious population reduction levels, hypothetically assuming the estimates were based on situations in which 100% of the village cattle were treated with fipronil and treatments occurred either 3, 6, or 12 times per year using the treatment schedules described by Poché et al. [34]. We summarized these results in the form of heat maps, as described above, except that isolines on the heat map surfaces represented all combinations of AFC and FOF that produced the efficacious sand fly population reduction levels reported by Stauch et al. [43], WHO [44], Fitzpatrick et al. [45], da Paixão Sevá et al. [46].

## Results

**Fig 2:**
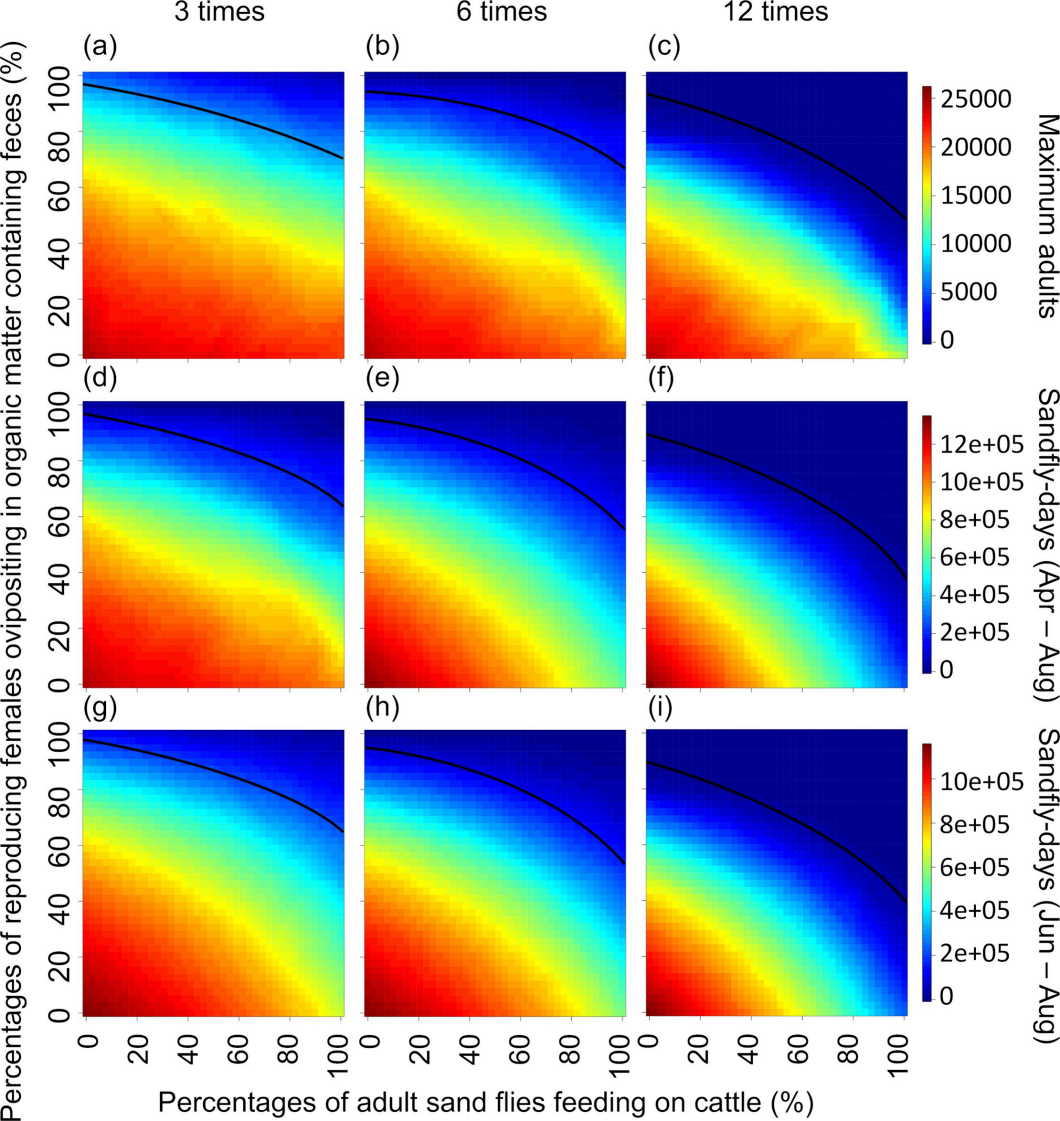
Heat maps representing how uncertainties associated with sand fly ecology with 100% of the cattle treated could affect ability to assess efficacy of the control schemes described by Poché et al. [34]. Efficacy (low/red to high/blue) is assessed in terms of maximum daily abundance of adult sand flies occurring during April through August, cumulative number of sand-fly-days during April through August, and cumulative number of sand-fly-days during June through August (rows), for sand fly control treatments applied to all cattle 3, 6, and 12 times per year (columns, see text for calendar dates of applications). Assuming a complete lack of prior knowledge, both the percentage of **a**dult sand flies **f**eeding on **c**attle (AFC, x-axis) and the percentage of reproducing **f**emales **o**vipositing in organic matter containing **f**eces from treated cattle (FOF, y-axis) could range from 0% to 100%. Black lines on heat map surfaces represent all combinations of AFC and FOF that produce the efficacies predicted by Poché et al. [34]. Percentages of surface areas above and below the lines represent probabilities that efficacies are higher or lower, respectively, than those predicted by Poché et al. [34].

Assuming a complete lack of prior knowledge regarding both AFC and FOF, simulation results indicate that uncertainty associated with the latter introduces more doubt into our ability to assess control efficacy than uncertainty associated with the former (Fig 2). (The shading on the heat maps in Fig 2 change relatively more along the y-axis for a given value on the x-axis than along the x-axis for a given value on the y-axis.) In the current simulations (100% cattle treated) for the 3-treatment scheme, the probabilities of achieving at least the ≈90% reductions in maximum sand fly abundance April-August (MAS), ≈83% reductions in cumulative sand-fly-days April-August (SDAA), and ≈85% reductions in cumulative sand-fly-days June-August (SDJA) previously predicted by Poché et al. [34] were ≈16%, ≈15%, and ≈15%, respectively (the percentages of the heat map surface areas above the black lines in Fig 2A, 2D and 2G, respectively). For the 6-treatment scheme, the probabilities of achieving at least the ≈95% MAS, ≈97% SDAA, and ≈97% SDJA reductions previously predicted by Poché et al. [34] were ≈16%, ≈15%, and ≈15%, respectively (Fig 2B, 2E and 2H). For the 12 treatment scheme, the probabilities of achieving at least the ≈99% MAS, ≈99% SDAA, and ≈99% SDJA reductions previously predicted by Poché et al. [34] were ≈22%, ≈31%, and ≈33%, respectively (Fig 2C, 2F and 2I).

**Fig 3:**
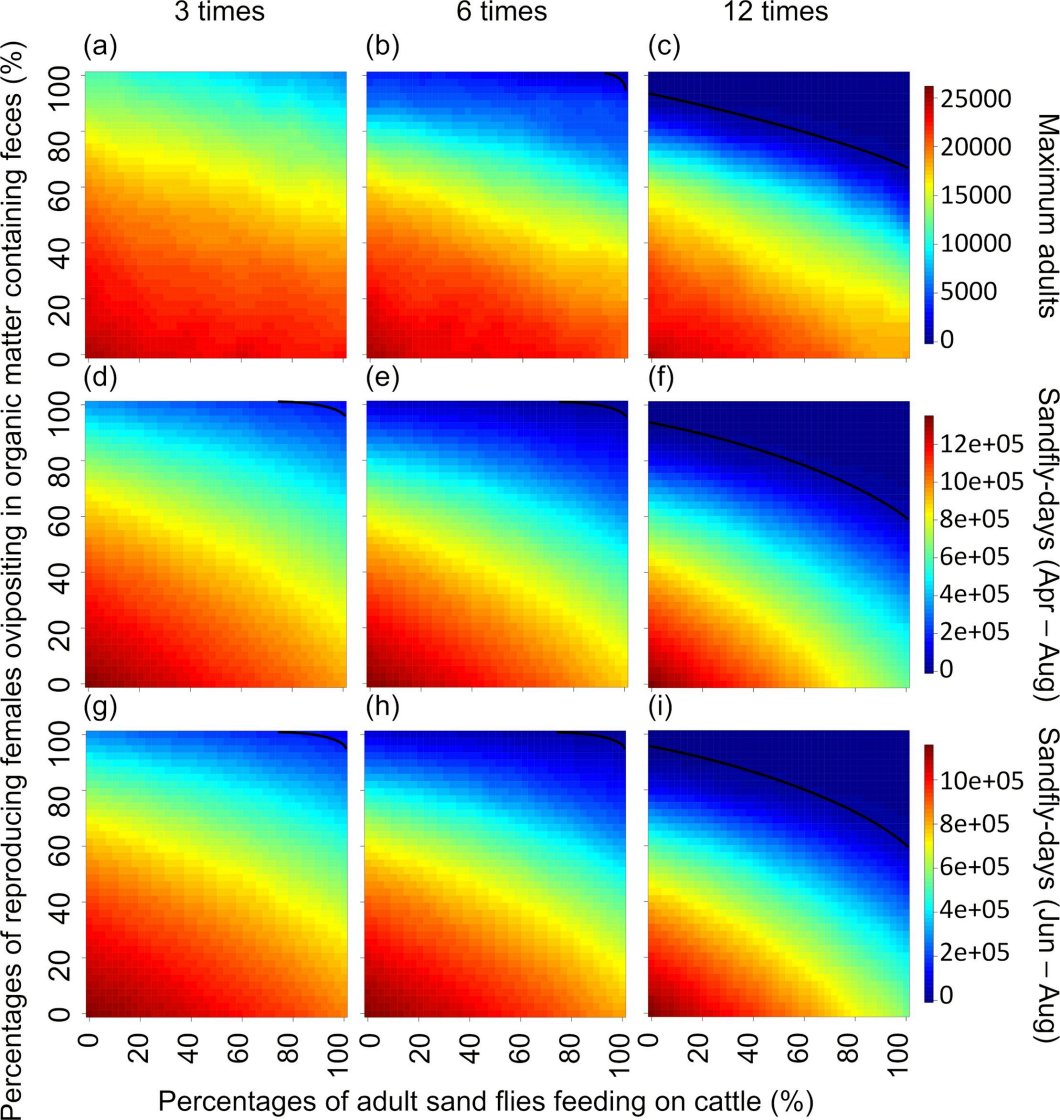
Heat maps representing how uncertainties associated with sand fly ecology with 66.1% of the cattle treated could affect ability to assess efficacy of the control schemes described by Poché et al. [34]. Efficacy (low/red to high/blue) is assessed in terms of maximum daily abundance of adult sand flies occurring during April through August, cumulative number of sand-fly-days during April through August, and cumulative number of sand-fly-days during June through August (rows), for sand fly control treatments applied to 66.1% of the cattle 3, 6, and 12 times per year (columns, see text for calendar dates of applications). Assuming a complete lack of prior knowledge, both the percentage of **a**dult sand flies **f**eeding on **c**attle (AFC, x-axis) and the percentage of reproducing **f**emales **o**vipositing in organic matter containing **f**eces from treated cattle (FOF, y-axis) could range from 0% to 100%. Black lines on heat map surfaces represent all combinations of AFC and FOF that produce the efficacies predicted by Poché et al. [34]. Percentages of surface areas above and below the lines represent probabilities that efficacies are higher or lower, respectively, than those predicted by Poché et al. [34].

With 66.1% of the cattle treated, for the 3-treatment scheme, the probabilities of achieving at least the ≈90% reductions in maximum sand fly abundance April-August (MAS), ≈83% reductions in cumulative sand-fly-days April-August (SDAA), and ≈85% reductions in cumulative sand-fly-days June-August (SDJA) predicted by Poché et al. [34] were ≈0%, ≈3%, and ≈3%, respectively (the percentages of the heat map surface areas above the black lines in Fig 3A, 3D and 3G, respectively). For the 6-treatment scheme, the probabilities of achieving at least the ≈95% MAS, ≈97% SDAA, and ≈97% SDJA reductions predicted by Poché et al. [34] were ≈1%, ≈3%, and ≈3%, respectively (Fig 3B, 3E and 3H). For the 12 treatment scheme, the probabilities of achieving at least the ≈99% MAS, ≈99% SDAA, and ≈99% SDJA reductions predicted by Poché et al. [34] were ≈19%, ≈22%, and ≈23%, respectively. (Fig 3C, 3F and 3I).

**Fig 4:**
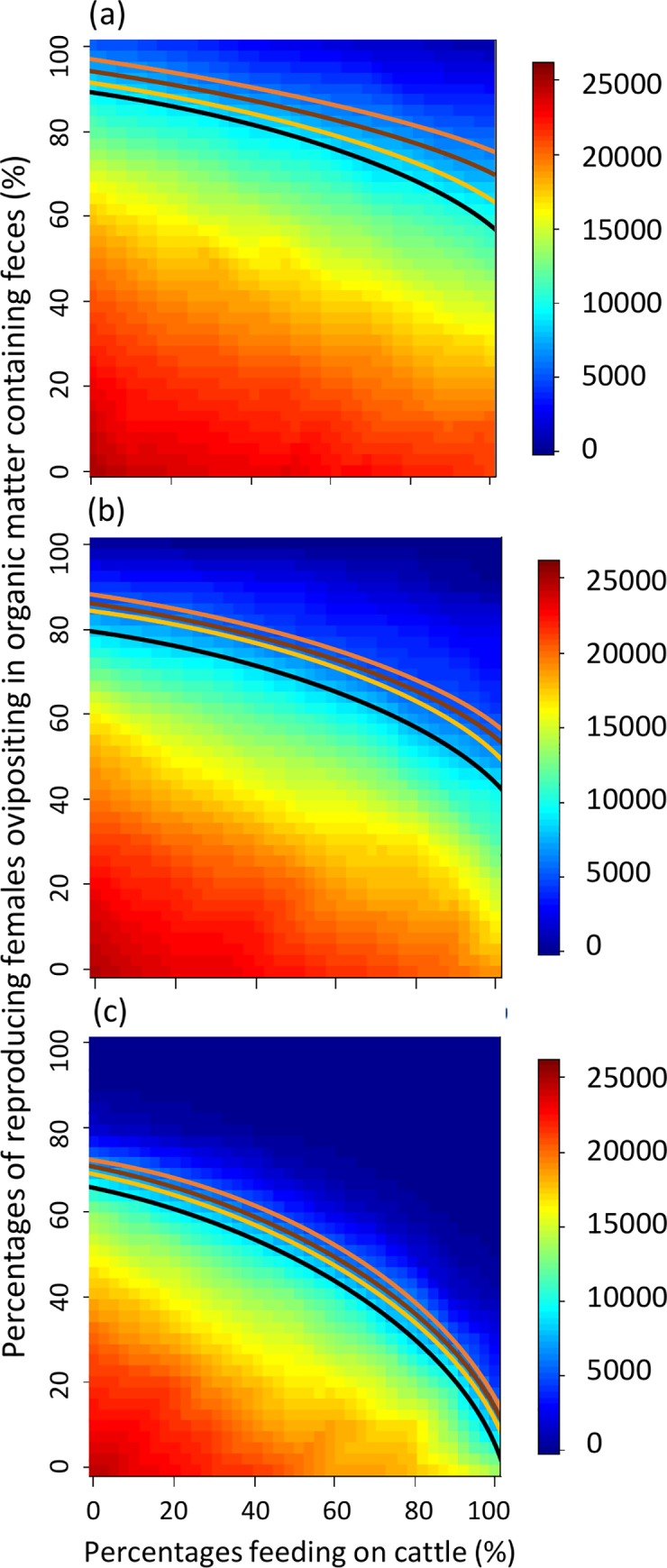
Heat maps representing how uncertainties associated with sand fly ecology with 100% of the cattle treated could affect ability to assess efficacy of the control schemes described by Stauch et al. [43], WHO [44], Fitzpatrick et al. [45], and da Paixão Sevá et al. [46]. Heat maps are interpreted as described in Fig 2. Black, orange, yellow, and brown lines on heat map surfaces represent all combinations of the percentage of adult sand flies feeding on cattle (x-axis) and the percentage of reproducing females ovipositing in organic matter containing feces from treated cattle (y-axis) that produce the sand fly populations reported as indicative of efficacious control by Stauch et al. [43], WHO [44], Fitzpatrick et al. [45], and da Paixão Sevá et al. [46], respectively. Percentages of surface areas above and below a given line represent probabilities that sand fly populations are higher or lower, respectively, than those reported as indicative of efficacious control by the authors of the corresponding study.

Application of these results (with 100% of the cattle treated) to the studies of Stauch et al. [43], WHO [44], Fitzpatrick et al. [45], da Paixão Sevá et al. [46] are summarized in Fig 4. This extension of our results is based on the hypothetical assumption that these studies were conducted in situations similar to those described by Poché et al. [34], and assumes, as above, a complete lack of prior knowledge regarding both AFC and FOF. With 100% of the cattle treated, for the 3-treatment scheme, the probabilities of achieving sand fly population reductions of at least 67–72% [43], 80% [44], 70–90% [45], and 75% [46] were ≈19–22% (the percentages of the heat map surface areas above the black line in Fig 4A), ≈14% (the percentages of the heat map surface areas above the orange line in Fig 4A), ≈4–21% (the percentages of the heat map surface areas above the yellow line in Fig 4A), and ≈17%, respectively. The analogous probabilities for the 6-treatment scheme were ≈32–36%, ≈27%, ≈13–33%, and ≈30% (Fig 4B), and for the 12-treatment scheme were ≈50–54%, ≈46%, ≈50–51%, and ≈48% (Fig 4C).

**Fig 5:**
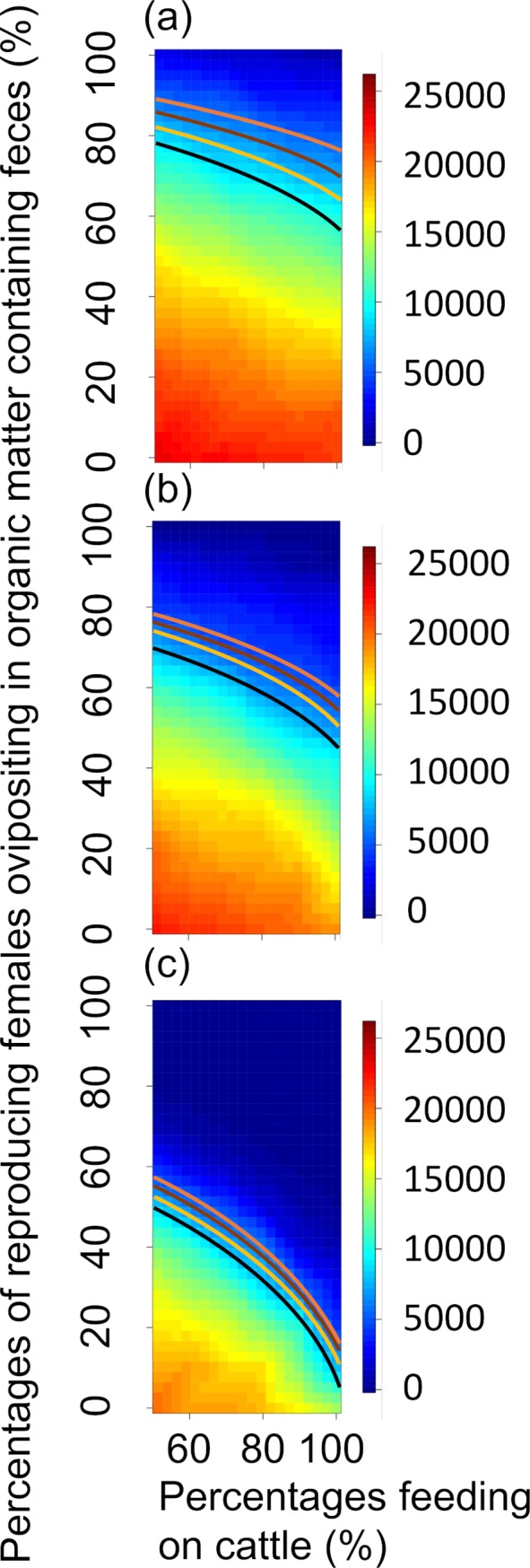
Heat maps representing how uncertainties associated with sand fly ecology with 100% of the cattle treated could affect ability to assess efficacy of the control schemes described by Stauch et al. [43], WHO [44], Fitzpatrick et al. [45], and da Paixão Sevá et al. [46]. Heat maps are interpreted as described in Fig 2. Note restriction of the scale on the x-axis compared to Fig 3, which represents prior knowledge about the percentage of adult sand flies feeding on cattle.

**Fig 6:**
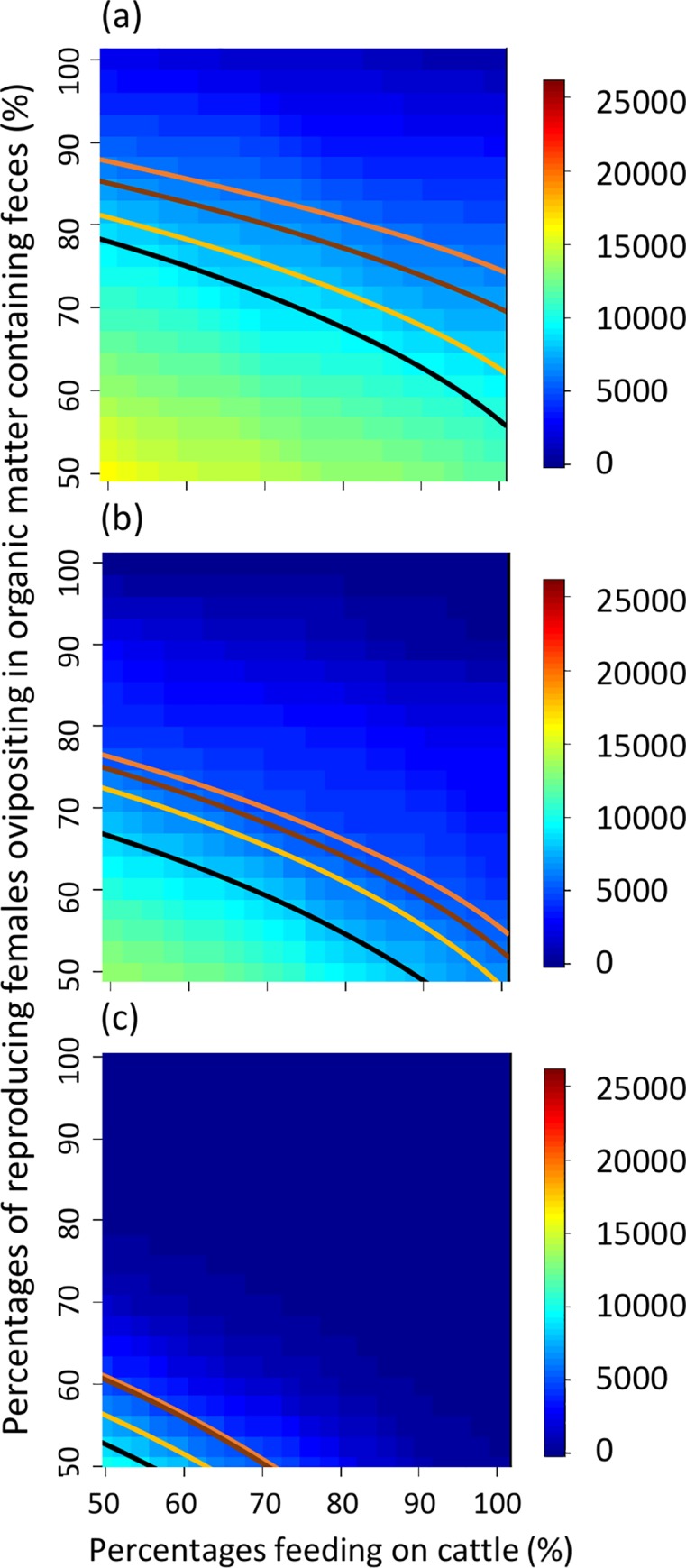
Heat maps representing how uncertainties associated with sand fly ecology with 100% of the cattle treated could affect ability to assess efficacy of the control schemes described by Stauch et al. [43], WHO [44], Fitzpatrick et al. [45], and da Paixão Sevá et al. [46]. Heat maps are interpreted as described in Fig 2. Note restriction of the scale on the y-axis compared to Figs 3 and 4, which represents prior knowledge about the percentage of reproducing females ovipositing in organic matter containing feces from treated cattle (in addition to prior knowledge about the percentage of adult sand flies feeding on cattle).

If we assume prior knowledge suggests AFC is >50%, probabilities of achieving the efficacious control levels of Stauch et al. [43], WHO [44], Fitzpatrick et al. [45], and da Paixão Sevá et al. [46] are increased to ≈22–31%, ≈15%, ≈8–25%, and ≈21% with the 3-treatment scheme (Fig 5A), to ≈40–42%, ≈32%, ≈15–40%, and ≈36% with the 6-treatment scheme (Fig 5B), to ≈62–65%, ≈58%, ≈52–63%, and ≈59% with the 12-treatment scheme (Fig 5C). If we assume prior knowledge suggests FOF also is >50%, the above probabilities are increased further to ≈44–53%, ≈31%, ≈14–50%, and ≈39% with the 3-treatment scheme (Fig 6A), to ≈75–81%, ≈61%, ≈31–75%, and ≈67% with the 6-treatment scheme (Fig 6B), and to ≈97%, ≈94%, ≈89–97%, and ≈94% with the 12-treatment scheme (Fig 6C).

## Discussion

Visceral leishmaniasis is a neglected vector-borne disease, with vector control being a key component in reducing disease transmission [47]. While IRS may have potential to control indoor sand fly populations, the sizable percentage of outdoor feeding *P*. *argentipes* pose considerable risk to Bihari villagers. *P*. *argentipes* abundance in Bihari villages is greatest during the months of June-August, when minimum daily temperatures are highest [12, 18], with many cases of VL being reported during April-August [48]. While bed net usage can protect against VL during warmer months [49], it is limited in Bihar because many villagers sleep outdoors [17, 50], and research indicates that bed net usage decreases in response to increased temperatures [51–53]. Hence, new vector control innovations are needed [54], particularly strategies that disrupt the sand fly life cycle and target outdoor *P*. *argentipes* populations. Use of the systemic insecticide fipronil administered orally to cattle shows great promise in this regard. We hasten to note, as Poché et al. previously stated [34], this form of treatment is meant to supplement rather than replace other forms of control, and its greatest value would be in areas where cattle density, and subsequently AFC, are relatively high. Further, pending a large-scale field trial, uncertainties in assessing the efficacy of fipronil-based control schemes remain, the effects of which we have analyzed in the current study.

Results of the current simulations with 100% of the cattle treated indicated changes in our assumptions regarding AFC and FOF values had a significant impact on our estimates of the success of fipronil-based control schemes. If we assumed greater dependence of *P*. *argentipes* on cattle (≥50% AFC and ≥50% FOF), simulation results suggested a maximum of 53, 81, 97% probability for success when treating 3, 6, and 12 times per year, respectively. If we assumed no prior knowledge of FOF (and ≥50% AFC), the probabilities of reducing sand flies decreased noticeably, with simulation results suggesting a maximum of 31, 42, and 65% probability of success when treating 3, 6, and 12 times per year. If we assumed no prior knowledge of either AFC or FOF, the probability of success decreased even more, with simulation results suggesting a maximum of 22, 36, and 54% when applied 3, 6, and 12 times per year.

These results emphasize the importance of having an appreciation for the interactions among cattle density, AFC, and FOF prior to initiation of control schemes. Prior research indicates that *P*. *argentipes* feeds opportunistically on village cattle and humans [7–12]. Logically, increased cattle abundance suggests an increased probability for *P*. *argentipes* to take blood meals from cattle and oviposit in cattle feces. Intuitively, increasing the frequency of control treatments increases the probability of successful control. However, frequent treatment is labor intensive, costly, and does not necessarily guarantee adequate control if cattle density is low. For example, consider the interaction of cattle density/AFC and FOF on likelihood of successful control in two villages in which treatments are applied 12 times per year. In the first village, cattle density/AFC is high (90% of adult sand flies feed on cattle). In the second village, cattle density/AFC is low (10%). To achieve reductions in sand fly populations below the benchmark VL epidemic threshold [43] in the first village, FOF could be as low as 15% (15% of oviposition occurring in organic matter containing feces from treated cattle). However, in the second village, FOF would need to exceed 65%. Additionally, if cattle density/AFC is high, treating at lesser application rates becomes more realistic. For example, if the first village were to be treated 6 and 3 times per year, FOF could be as low as 40% and 55%, respectively. Thus, even if the first village was treated only 3 and 6 times per year, relative to the 12 treatments performed in the low cattle density/AFC village, it would still require fewer FOF and thus have a higher probability of successfully controlling the sand fly abundance. Hence, managers should weigh the cost-benefits of treating at increased or reduced frequencies. Obviously treating at reduced rates would be more economical. However, reduced AFC will require more frequent treatments. Future research should consider developing a cattle density/AFC threshold below which fipronil cattle treatment would be ill-advised.

Since simulation results suggest that larval mortality has greater impact on *P*. *argentipes* populations than adult mortality, fipronil application would be more efficacious in areas with greater FOF. Several studies have indicated phlebotomine sand fly sensitivity to fipronil [26, 29–32], with *P*. *argentipes* larvae being particularly vulnerable [32]. Poché et al. recorded 100% larval mortality up to 21-days post treatment at all fipronil concentrations they administered to cattle (*Bos taurus*) [32]. This sensitivity of larvae to fipronil is reflected in our simulation results. Unfortunately, there is a deficit of explicit oviposition site data [55], which would be invaluable in predicting the success of fipronil control schemes. Although extensive investigations of natural oviposition sites have been conducted, only a few isolated studies in Italy [56–58] and in Panama [59] have yielded significant numbers of larvae, and larval numbers from field studies are almost universally low [55, 60]. Therefore, we would argue that studies investigating oviposition sites of *P*. *argentipes* by novel means are of paramount importance.

Thus, although the indiscriminate blood-feeding behavior of *P*. *argentipes* suggests that AFC values are a byproduct of cattle abundance, our lack of knowledge about oviposition sites makes it difficult to draw the same conclusion regarding FOF values with confidence. However, while field collections of immature sand flies (eggs, larvae, pupae) typically yield small numbers, they are often found in proximity to village cattle and cattle feces [35, 61–63], with cattle feces being the overwhelming majority of the organic material present within these villages. A benefit of fipronil treatment is that it remains in the system of the treated animal and therefore residual fipronil is excreted in feces daily over several weeks [32, 34], meaning that freshly excreted feces can yield control of newly-hatched sand fly larvae several weeks after treatment is performed. This coupled with the extended half-life of fipronil [64], suggests the potential efficacy of fipronil cattle treatment in larval control.

Another reason for the increased efficacy against sand fly larvae is likely the fact that the majority of ingested fipronil is excreted in feces [65]. Fipronil residues in blood result in efficacy against adult sand flies with a whole-blood half-life in rats estimated to be 6.2–8.3 days [64]. Fipronil is lipophilic so additional residue is found in fatty tissues as well [66]. It is important to note that some fipronil residues are present in milk of treated animals. India is the highest producer of milk in the world, estimated to produce >176 million tons of milk during 2017 [67]. Therefore, dangerously high residue levels in milk would raise significant concerns. A study aimed at evaluating metabolism and excretion of fipronil in ruminants, found that goats orally administered 10 mg fipronil/kg body weight 2x per day, for 7-days, excreted ~61%, of the administered fipronil through feces [65]. Approximately 7.4% of the fipronil was recovered from tissues, particularly fat, with the remainder being in urine (6.6%) and milk (1.3%) [65]. This treatment frequency was much higher than those simulated during our treatment schemes and the dose was ≈20x higher than what we simulated (0.5 mg/kg). This suggests that fipronil cattle treatment would result in minimal fipronil residues in milk. It should additionally be noted, as a worst-case scenario, that cases of deliberate self-poisoning with large quantities of fipronil are suggested to be manageable [68]. Additional laboratory studies with cattle and domestic buffalo would be useful in determining explicit fipronil residue levels at various timepoints post-treatment. Further research regarding the residue levels of fipronil in cattle milk is in progress and will aid in determining the frequency at which fipronil treatment should be implemented.

Further, fipronil would likely have no negative impacts on the health of cattle because of the low application rate (3–12 times per year) and the low dosage administered (0.5 mg/kg bodyweight). Prior researchers presented buffalo cattle orally with fipronil at a rate of 0.5 mg/kg bodyweight/day for 21 consecutive days [69]. Even at this elevated, ill-advised application rate, only mild and moderate signs of toxicity were observed, and symptoms ceased completely within days of terminating fipronil exposure. Contrarily, less frequent fipronil application has the potential to improve the health of bovines by removing ectoparasites. Cattle are often heavily infested with ectoparasites such as ticks in India, which in addition to leading to disease transmission causes extensive harm to livestock production and health [70]. Prior research indicates that oral fipronil can remove ticks [23] and fleas [24, 26] infesting rodents under laboratory and field conditions. Poché et al. [32] noted a significant decrease in tick numbers on cattle treated orally with a single dose of 0.5 mg/kg fipronil under pen conditions. Explicit research should be conducted to determine the potential risks and benefits to cattle resulting from use of orally administered fipronil.

We should note that we assumed that no insecticide resistance occurred during our treatment schemes. Insecticide resistance is always of concern when applying compounds in the field and research relating to fipronil resistance in sand flies is lacking. However, there is little direct evidence suggesting that resistance to fipronil occurs [22, 71]. Research involving other arthropod vectors, such as the cat flea (*Ctenocephalides felis*), suggest no fipronil resistance in laboratory and field strains [72, 73]. The relative infrequency of treatment application, in conjunction with the fact that this treatment targets two life stages, should limit the relative risk of resistance occurring. However, we encourage researchers to monitor treated sand fly populations and to continue to evaluate the potential for insecticide resistance to occur.

In summary, our results place previous assessments of the potential efficacy of fipronil-based sand fly control [34] within a broader management perspective. After quantifying current uncertainties associated with sand fly ecology under conditions representative of villages in Bihar, our simulations suggest that fipronil orally administered to village cattle has the potential to reduce sand fly abundance well below benchmark VL epidemic thresholds [43]. However, treatment efficacy likely will vary among villages depending on availability and characteristics of hosts (treated cattle versus untreated cattle plus alterative hosts) and oviposition sites (organic material containing feces from treated cattle versus organic material not containing feces from treated cattle). In relative terms, efficacy should be high even with infrequent treatment in villages with high cattle densities and the capability to treat a high percentage of the cattle. In villages with lower cattle densities and/or the inability to treat a high percentage of the cattle, efficacy should decline even with frequent treatments. These simulated trends are robust with regard to the (parametric) uncertainties associated with AFC and FOF. The largest source of uncertainty affecting assessment of efficacy is the uncertainty associated with FOF. That is, in practical terms, ability of a specific treatment scheme to reduce sand fly abundance below benchmark VL epidemic thresholds in a specific village depends heavily on the availability of fipronil-free oviposition sites.

In conclusion, we would suggest that uncovering additional ecological uncertainties relating to locations of oviposition sites would aid in further parameterizing the model and the development of supplemental control methods [74]. The results of our simulations suggest that this model may aid in predicting the outcome of fipronil cattle treatment under a variety of ecological scenarios representative of the villages in Bihar, which could potentially contribute to the planning and execution of a largescale field trial. The use of our model to better understand the relationship between sand fly control schemes and the sand fly life cycle may prove useful in evaluating and implementing supplemental vector control strategies on the Indian subcontinent and in other VL-infected regions.

## Supporting information

S1 File Appendix. (DOCX)
