# Supplementary material for: Visceral leishmaniasis on the Indian Subcontinent: Efficacy of fipronil-based cattle treatment in controlling sand fly populations is dependent on specific aspects of sand fly ecology
Source: PLoS Negl Trop Dis. 2020 Feb 18;14(2):e0008011. doi: 10.1371/journal.pntd.0008011 (PMC7048295; doi:10.1371/journal.pntd.0008011)
Supplement: S1 File — (DOCX) [file pntd.0008011.s001.docx]

**Appendix**

In this appendix we present verification that our current model produces the same results as the model of Poché et al. 2016 under the conditions that they simulated (assuming treatments were applied to 100% of the village cattle, 50% of the adult sand flies fed on cattle, and 90% of the reproducing females oviposited in organic matter containing feces from treated cattle). Specifically, we compared the two models with regard to (a) maximum number of adult sand flies, (b) cumulative number of sand-fly-days during April through August, and (c) during June through August, assuming (1) no sand fly control treatments are applied (App. Figure 1), (2) assuming sand fly control treatments are applied 3 times per year (App. Figure 2), (3) assuming sand fly control treatments are applied 6 times per year (App. Figure 3), and (4) assuming sand fly control treatments are applied 12 times per year (App. Figure 4). We used T-tests based on results of 10 replicated stochastic (Monte Carlo) simulations to compare outputs of the two models. We summarize results of these comparisons below.

**No sand fly control treatments applied**

Maximum number of adult sand flies:

1. no statistically significant variations (mean = 24683.0202, median = 24687, standard error = 9.8315; t Stat = 0.1086, p-value = 0.9136)
2. no statistically significant difference from the results of Poche et al. [1] (t Stat = -0.4652, p-value = 0.6422)

Cumulative number of sand-fly-days during April through August:

1. no statistically significant variations (mean = 1,327,640, median = 1,327,555, standard error = 199.0008; t Stat = -0.5910, p-value = 0.5551)
2. no statistically significant difference from the results of Poche et al. [1] (t Stat = -0.3857, p-value = 0.7001)

Cumulative number of sand-fly-days during June through August:

1. no statistically significant variations (mean = 1,141,360, median = 1,141,298, standard error = 162.1772; t Stat = -0.0065, p-value = 0.9949)
2. no statistically significant difference from the results of Poche et al. [1] (t Stat = -0.1371, p-value = 0.8911)

**Sand fly control treatments applied 3 times per year**

Maximum number of adult sand flies:

mean = 5,323.67, median = 5,440, standard error = 138.91; no statistically significant difference from the results of Poche et al. [1] (t Stat = -0.8221, p-value = 0.4572)

Cumulative number of sand-fly-days during April through August:

mean = 212,397.3, median = 214,989, standard error = 5,980.53; no statistically significant difference from the results of Poche et al. [1] (t Stat = 0.0235, p-value = 0.9824)

Cumulative number of sand-fly-days during June through August:

mean = 172,168.3, median = 174,713, standard error = 4,633.522; no statistically significant difference from the results of Poche et al. [1] (t Stat = 0.3972, p-value = 0.7115)

**Sand fly control treatments applied 6 times per year**

Maximum number of adult sand flies:

mean = 2,799.667, median = 2,816, standard error = 43.7810; no statistically significant difference from the results of Poche et al. [1] (t Stat = 0.0046, p-value = 0.9966)

Cumulative number of sand-fly-days during April through August:

mean = 64,511, median = 64,624, standard error = 350.7026; no statistically significant difference from the results of Poche et al. [1] (t Stat = -0.3153, p-value = 0.7683)

Cumulative number of sand-fly-days during June through August:

mean = 54,627.33, median = 174,713, standard error = 424.3349; no statistically significant difference from the results of Poche et al. [1] (t Stat = 0.8221, p-value = 0.4572)

**Sand fly control treatments applied 12 times per year**

Maximum number of adult sand flies:

mean = 1.6667, median = 1, standard error = 1.2019; no statistically significant difference from the results of Poche et al. [1] (t Stat = 0.0615, p-value = 0.9539)

Cumulative number of sand-fly-days during April through August:

mean = 20, median = 13, standard error = 14.0119; no statistically significant difference from the results of Poche et al. [1] (t Stat = 0.9093, p-value = 0.9324)

Cumulative number of sand-fly-days during June through August:

mean = 3.6667, median = 1, standard error = 2.6667; no statistically significant difference from the results of Poche et al. [1] (t Stat = -0.7528, p-value = 0.4935)

**Appendix References**

1. Poché DM, Grant WE, Wang H-H. Visceral leishmaniasis on the Indian subcontinent: Modelling the dynamic relationship between vector control schemes and vector life cycles. PLoS Neglected Tropical Diseases. 2016;10(8):e0004868.

**
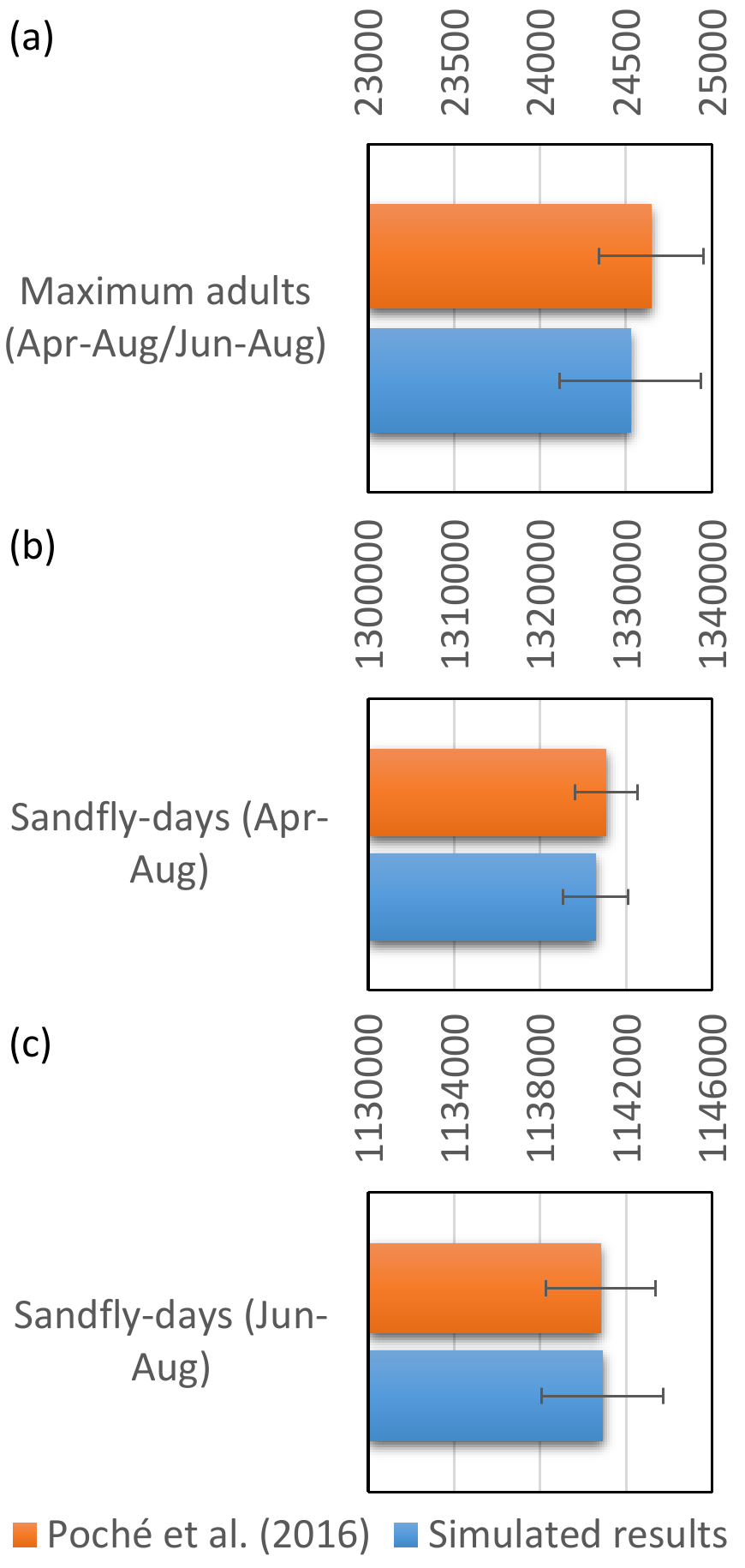
**

Appendix Figure 1. Comparison of results of the current model with those of the model of Pouché et al. [1] with regard to (a) maximum number of adult sand flies, (b) cumulative number of sand-fly-days during April through August, and (c) during June through August, assuming no sand fly control treatments are applied.


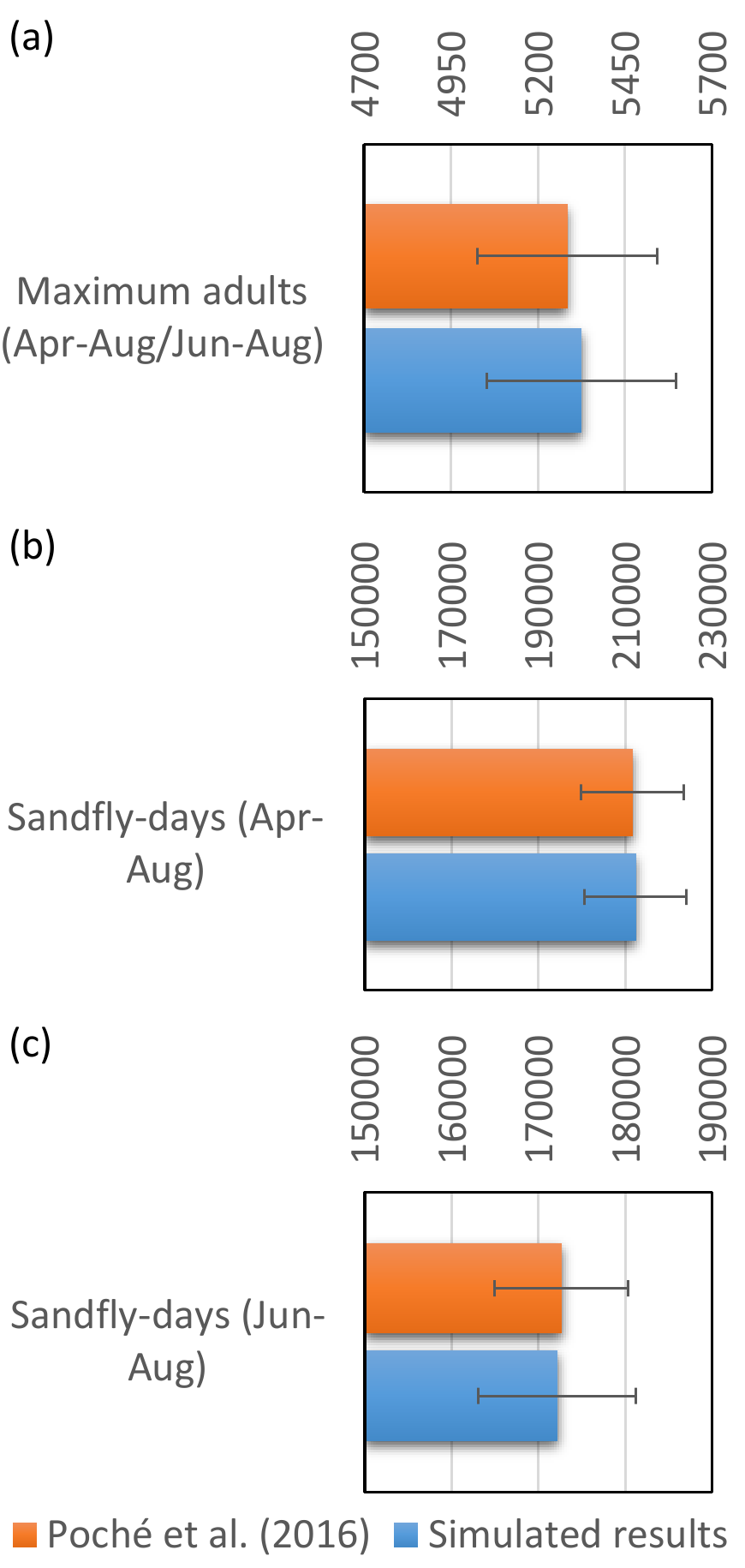


Appendix Figure 2. Comparison of results of the current model with those of the model of Pouché et al. [1] with regard to (a) maximum number of adult sand flies, (b) cumulative number of sand-fly-days during April through August, and (c) during June through August, assuming sand-fly control treatments are applied 3 times per year.


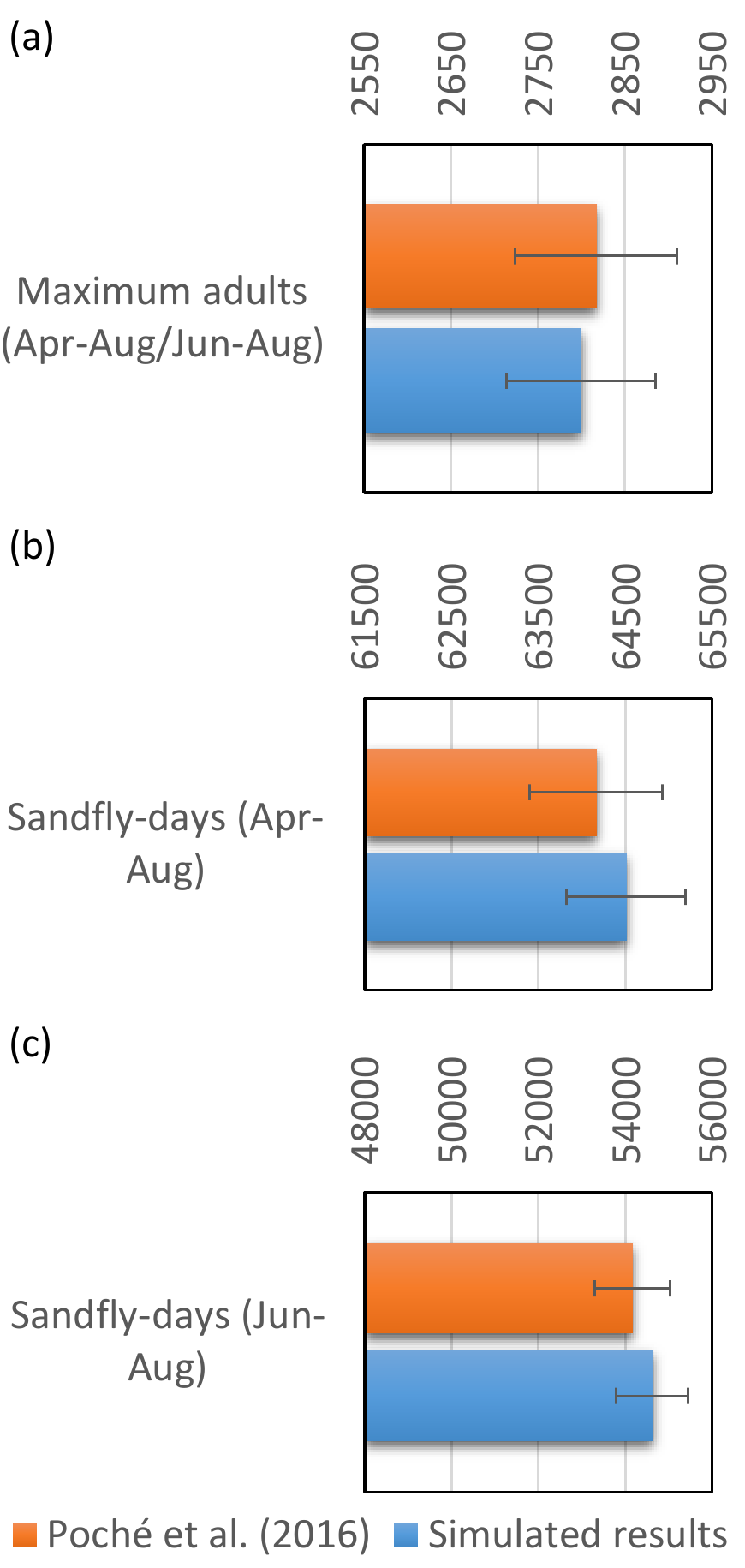


Appendix Figure 3. Comparison of results of the current model with those of the model of Pouché et al. [1] with regard to (a) maximum number of adult sand flies, (b) cumulative number of sand-fly-days during April through August, and (c) during June through August, assuming sand-fly control treatments are applied 6 times per year.


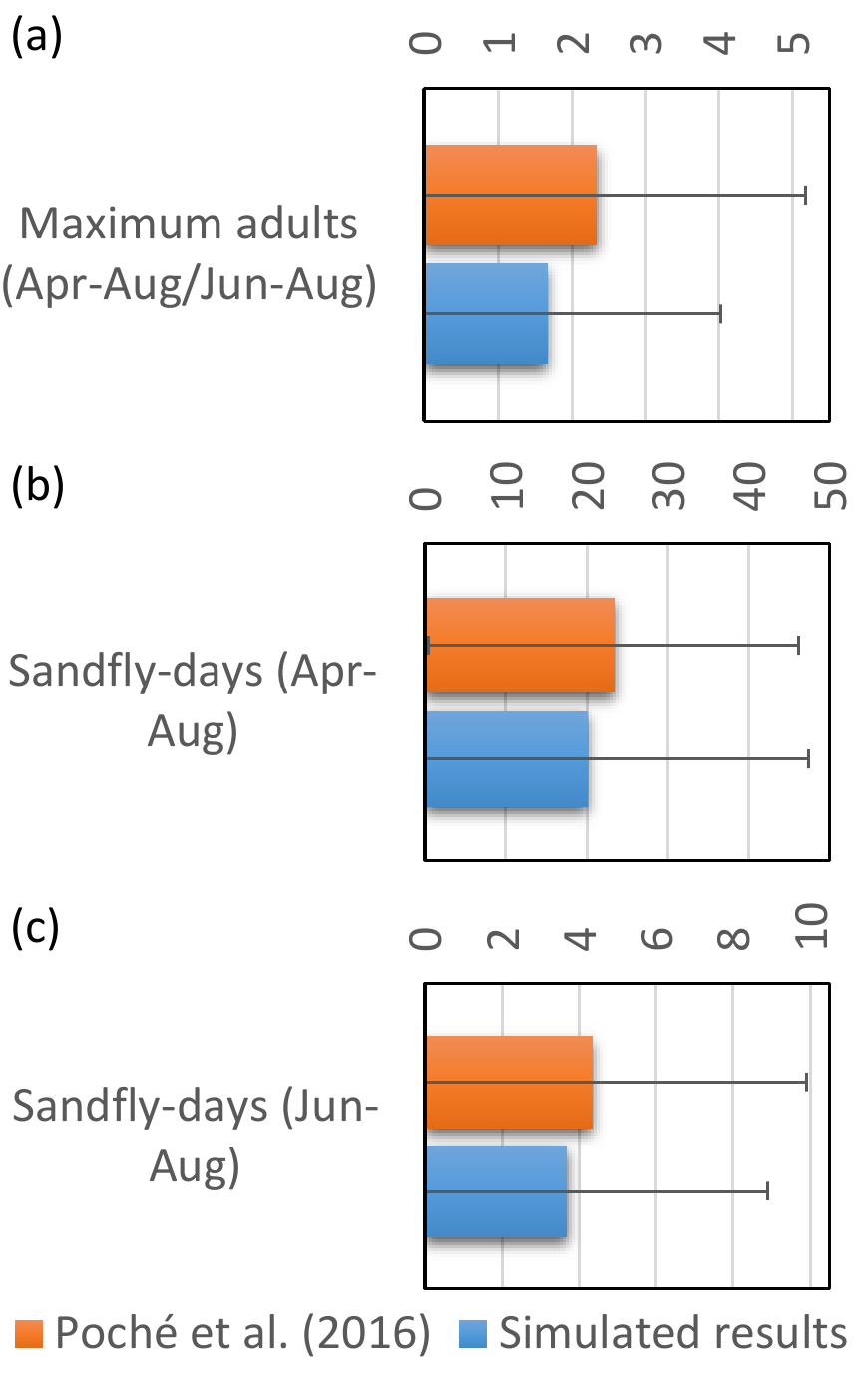


Appendix Figure 4. Comparison of results of the current model with those of the model of Pouché et al. [1] with regard to (a) maximum number of adult sand flies, (b) cumulative number of sand-fly-days during April through August, and (c) during June through August, assuming sand-fly control treatments are applied 12 times per year.
